# Complications and Outcomes of the Nuss Procedure in Adult Patients: A Systematic Review

**DOI:** 10.7759/cureus.35204

**Published:** 2023-02-20

**Authors:** Muzammil Akhtar, Daniel I Razick, Ali Saeed, Osamah Baig, Rafaay Kamran, Ubaid Ansari, Zahra Sajid, Joseph E Rahman

**Affiliations:** 1 Surgery, California Northstate University College of Medicine, Elk Grove, USA; 2 Internal Medicine, William Carey University College of Osteopathic Medicine, Hattiesburg, USA; 3 Ophthalmology, Lake Erie College of Osteopathic Medicine, Erie, USA; 4 Molecular Biology, University of California Berkeley, Berkeley, USA; 5 Internal Medicine, California Northstate University College of Medicine, Elk Grove, USA; 6 Biology, Cosumnes River College, Elk Grove, USA; 7 Cardiology, PIH (Partners in Health) Health, Whittier, USA

**Keywords:** outcomes, complications, pectus excavatum, adults, mirpe, nuss procedure

## Abstract

Pectus excavatum is a congenital chest wall deformity, commonly identified in early childhood, creating a “sunken chest” appearance. Over time, the deformity can worsen, thus impacting cardiopulmonary function and creating significant body image disturbance in patients. The Nuss procedure is a minimally invasive technique in which a curved steel bar is introduced underneath the sternum through small bilateral thoracic incisions to correct the deformity. Most studies regarding the procedure to date focus on outcomes and complications in pediatric patients, however, few studies discuss these results in adult patients. This systematic review aims to analyze common complications and outcomes in patients over the age of 18 who have not undergone any prior intervention for pectus excavatum. The most common complications experienced in adult patients were displacement of the implanted steel bar, infection of the surgical site, pneumothorax, pleural effusion, and chronic postoperative pain. Reoperation was common in patients with a displacement of the bar, chronic pain, and bleeding. Additionally, adult patients routinely required a higher number of steel bars to be placed to correct the deformity. Despite evidence that the rate of complications increases with age, the majority of adult patients in our included studies were satisfied with the outcome of the procedure with indications of improved self-image and reduced preoperative symptoms such as dyspnea on exertion, palpitations, chest pain, and depression.

## Introduction and background

Pectus excavatum (PE) is a congenital chest wall deformity in which the sternum is posteriorly depressed, creating a caved-in appearance classically described as a “sunken chest.” PE is approximately five times more likely to occur in male as compared to female patients. Though the exact cause of PE remains unknown, disturbances in sternal growth and biomechanical factors inducing abnormal stress on surrounding cartilage seem to play a role in pathogenesis. Additionally, PE is commonly associated with heritable connective tissue disorders such as Marfan syndrome and Ehlers-Danlos syndrome [1-2].

PE is commonly identified in early childhood and many patients experience an increase in sternal depression with age, prompting the need for surgical intervention. PE has traditionally been thought to be primarily a cosmetic defect, however, many studies report gradually worsening cardiopulmonary defects in PE patients, with significant improvement of symptoms after surgical repair, indicating the anatomic abnormality as the potential root cause of problems. Common symptoms of PE include progressive fatigue with mild exercise, chest pain, wheezing, dizziness, palpitations, and an inability to keep up with peers [1,3-4].

Surgical repair of PE was first proposed by Ravitch in 1949 who employed an open technique that requires bilateral resection of the involved costal cartilage to allow for a transverse wedge osteotomy of the sternum. Opposite edges of the osteotomy are then rejoined, allowing for the sternum to rise and for stabilization without any implants [5]. Minor modifications to the procedure have been made since its development, such as the implantation of metal struts to provide additional stabilization in certain cases, however, the overall procedure has remained essentially the same. A minimally invasive procedure was later introduced by Nuss et al. in 1998 in which a curved steel bar is introduced underneath the sternum through small bilateral thoracic incisions. The bar is then flipped to position it where the convexity faces anteriorly, thereby correcting the PE deformity. A subsequent procedure is performed when the deformity is permanently corrected, after approximately two years, in which the bar is removed [6]. Many modifications have been made to the minimally invasive procedure described by Nuss such as the use of multiple bars, shorter bars, forced sternal elevation, bilateral stabilizers, and bilateral thoracoscopy [7]. Major indications for the Nuss procedure include the progression of cardiopulmonary symptoms, a Haller index > 3, and significant body image disturbance [1].

The magnetic mini-mover procedure (3MP) is a recently conceived, minimally invasive method to treat PE still undergoing trials. In the procedure, a titanium-enclosed magnet is implanted into the sternum, and an external brace housing another magnet is placed on the anterior chest. These magnets apply a gradual outward force on the sternum, allowing for the correction of the PE deformity. In a trial of 15 adolescent patients, despite eight patients being satisfied with the results, seven patients experienced fatigue fractures of implanted titanium cables, bringing into question the efficacy of the procedure [8]. Though the purpose of this method was to limit pain experienced by patients who would otherwise have undergone the Ravitch or Nuss repair, advancements in pain management make the latter two better options for treating PE deformities.

The majority of patients undergoing the Nuss procedure are under the age of 18, however, some patients may show the development of symptoms and progression of the deformity at a later age, indicating the need for surgery in adulthood. The majority of studies regarding the Nuss procedure to date focus on outcomes and complications in pediatric populations of patients under the age of 18. However, few studies assessing the efficacy of the Nuss procedure in adult populations exist, and it is our aim in this review to explore common complications and outcomes in adult patients over the age of 18 who have not undergone any prior intervention for PE. The majority of studies in adult patients suggest favorable results with reported outcomes comparable to those in pediatric populations.

## Review

Methods

A literature search for this systematic review was performed on PubMed starting on January 11, 2023. Our search strategy was the following: (“Nuss”[Title/Abstract] AND “outcome”[Title/Abstract]), (“Nuss”[Title/Abstract] AND “complication”[Title/Abstract]), (“Nuss”[Title/Abstract] AND “failure”[Title/Abstract]), (“Nuss”[Title/Abstract] AND “adult”[Title/Abstract]), (“MIRPE”[Title/Abstract] AND “outcome”[Title/Abstract]), (“MIRPE”[Title/Abstract] AND “complication”[Title/Abstract]), (“MIRPE”[Title/Abstract] AND “failure”[Title/Abstract]), and (“MIRPE”[Title/Abstract] AND “adult”[Title/Abstract]).

The exclusion criteria were the following: articles in a different language, case reports, articles detailing surgical techniques, review articles, articles in which patients underwent prior surgical intervention for PE, patient population less than 18 years old, patient population with less than or equal to 10 patients, articles with incomplete data, and non-human studies. We included articles in which the Nuss procedure was performed in adult patients over the age of 18 years and in which the outcomes and complications were discussed [8-17].

**Figure 1:**
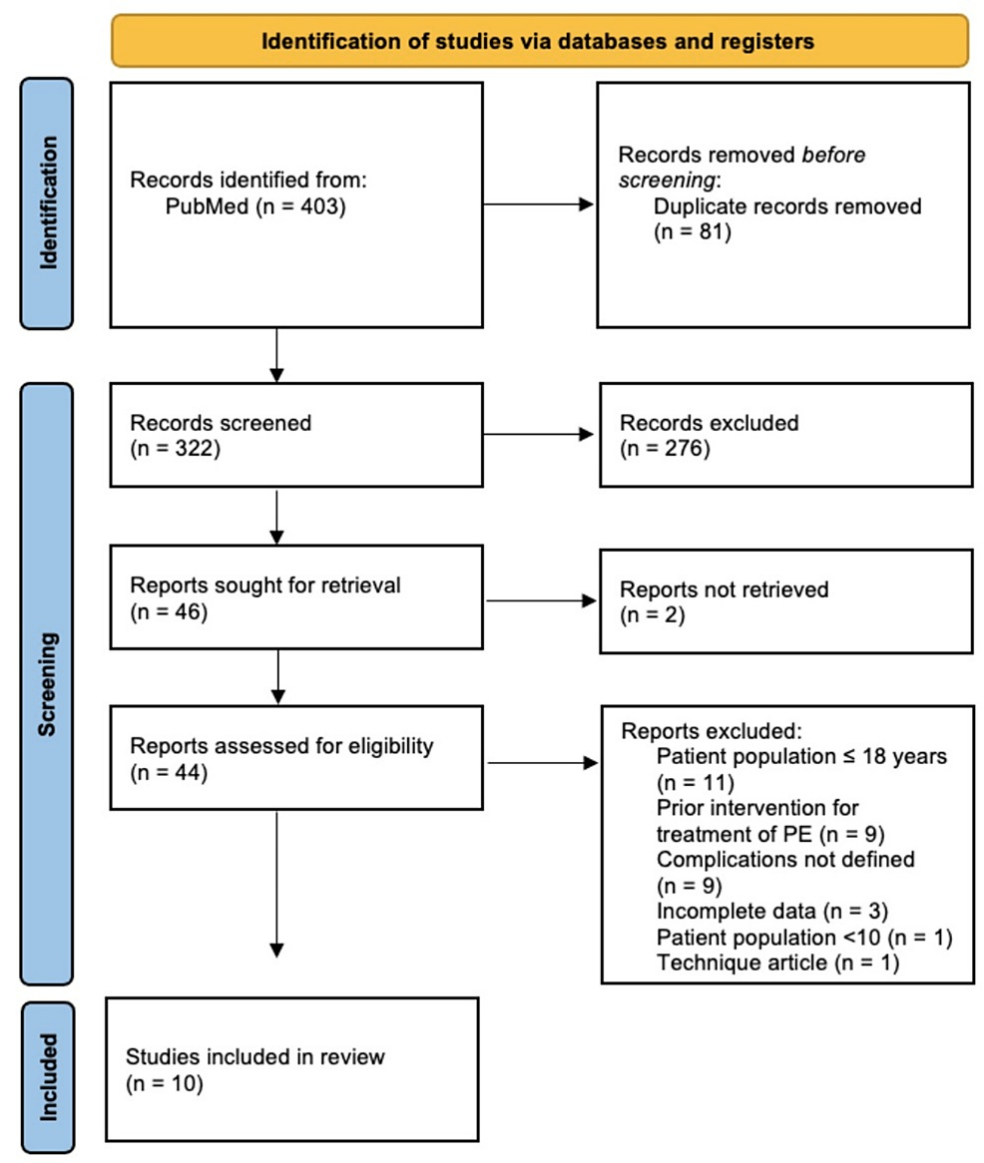
PRISMA diagram illustrating the article selection process PRISMA: Preferred Reporting Items for Systematic Reviews and Meta-Analyses

This search yielded 403 studies out of which 81 duplicates were removed. Screening of the title and abstract of the remaining 322 studies resulted in 276 being excluded due to irrelevance to the topic of outcomes and complications in adult patients undergoing the Nuss procedure. A full-text assessment was done of 44 studies, out of which 34 were excluded based on our established exclusion criteria. The 10 remaining studies were included in this systematic review. Figure 1 illustrates our article selection process, according to the Preferred Reporting Items for Systematic Reviews and Meta-Analyses (PRISMA) guidelines, used in our study. Identification of studies was done with the consensus of two authors and when an agreement could not be reached, a third author was consulted.

The following data were extracted from all 10 included articles: first author, article title, time range of the study, study type, number of patients, gender of patients, age of patients, indications for surgery, preoperative symptoms, Haller index, surgical technique, operation time, length of postoperative hospital stay, number of bars used, and all postoperative and perioperative complications. Data were extracted by two authors independently and differences were resolved through extensive discussion.

Results

**Figure 2:**
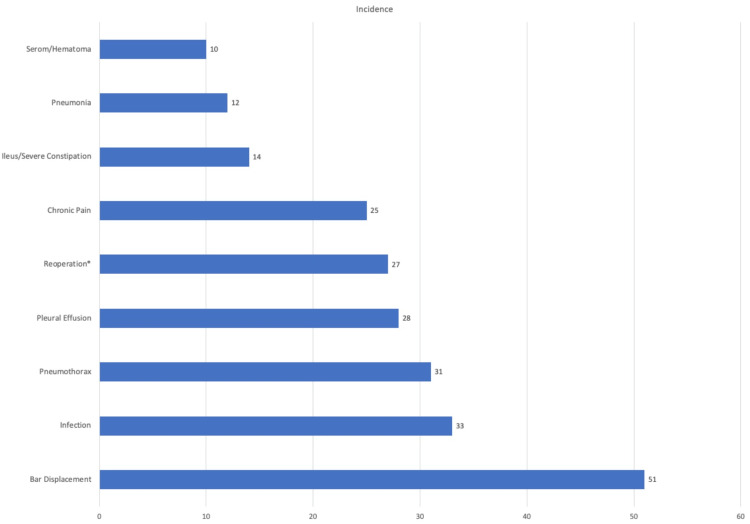
Complications occurring in five or more instances * = reoperation due to bleeding, removal of the bar, or chronic pain

A total of 1135 patients were included across the 10 studies in this systematic review, with 943 (83.1%) male and 192 (16.9%) female patients. The age of all patients ranged from 18 to 72 years old with the majority of patients being less than the age of 40 years (Table 1). Complications that occurred in five or more cases were as follows: displacement of the implanted steel bar in 51 (4.5%) patients, infection of the surgical site in 33 (2.9%) patients, pneumothorax in 31 (2.7%) patients, pleural effusion in 28 (2.5%), and chronic pain in 25 (2.2%) patients. Reoperation was required in 27 (2.4%) patients for a variety of reasons including bleeding, removal of the bar due to chronic pain, and displacement of the bar (Figure 2). Three patients required conversion to a modified Ravitch procedure due to severe asymmetry of the chest, which could not be corrected simply with a Nuss procedure. The number of steel bars used to correct the PE deformity was as follows: one bar in 561 patients (49.4%), two bars in 436 patients (38.4%), three bars in 137 patients (12.1%), and four bars in one patient (0.1%) (Table 1).

**Table 1:** Selected study characteristics, complications, and outcomes Age in Years, Preoperative Haller Index, Operation Time in Minutes, and Length of Hospital Stay are reported as “mean ± standard deviation (range)” when available unless otherwise noted * = median; † = interquartile range; NR = not reported

Author	Number of Patients (Male/Female)	Age in Years	Preoperative Haller Index	Operation Time in Minutes	Length of Hospital Stay in Days	Number of Bars (Incidence)	Complications and Outcomes (Incidence)
de Loos et al. (2021) [8]	55 (48/7)	32* (27-38)† Range: 25-47	3.6* (3-4.3)†	35* (30-45)†	5* (4-6)	1 bar (47), 2 bars (8)	Bar displacement (2), bar removal within three years due to chronic pain (4), bar removal within one year due to unmentioned reason (1), pneumonia (2), chronic pain without bar removal (4)
Erşen et al. (2016) [9]	236 (216/20)	23.2 (18-45)	4.4 (3.3-11)	44.4 (25-90)	4.92 ± 2.81 (3-21)	1 bar (200), 2 bars (36)	Bar displacement (12), cardiac injury (1), thoracic outlet syndrome (1), wound infection (4), pneumonia (1), sternotomy/thoracotomy (1), pneumothorax (7), pleural effusion (2), prolonged pain (4), regression of PE after bar removal (1)
Hebra et al. (2006) [10]	30 (23/7)	23 (18-32)	4.4	60-120 minutes in 60% of cases	NR	1 bar (25), 2 bars (5)	Seroma (3), bar displacement (2), pneumothorax (2), infection (1), stabilizer bar fracture (1), conversion to modified Ravitch repair (2)
Jaroszewski et al. (2016) [11]	266 (196/70)	33.18 (18-72)	5.71 (2.5-26.7)	116.7 (60-224)	NR	1 bar (1), 2 bars (148), 3 bars (116), 4 bars (1)	Bar displacement (12), infections (3), pneumonia (6), ileus/severe constipation (14), pleural effusion (12), pneumothorax (2), pulmonary embolism (2), urinary tract infection (6), reoperation for bleeding (3)
Kim et al. (2005) [12]	12 (all male)	27 ± 10.2 (20-52)	4.74 ± 1.56 (3-9.7)	127.3 ± 44.9	10 ± 8.5 (4-40)	1 bar (1), 2 bars (11)	Pleural effusion (1), wound infection due to stabilizer (3), chest pain > 6 months postoperation (6), bar displacement (4), reoperation (6), conversion to Ravitch procedure (1)
Lo et al. (2020) [13]	223 (193/30)	26.7	4.04	81.9	6.4	1 bar (21), 2 bars (181), 3 bars (21)	Pneumonia (1), pleural effusion (4), bar displacement requiring reoperation (9), wound infection (2), prolonged pain > 6 months (1)
Muhammad (2014) [14]	22 (18/4)	26.1	3.08	69.5	8.64	1 bar (all)	Pneumothorax (3), wound seroma (2), breakage of stabilizer (1)
Sacco Casamassima et al. (2016) [15]	98 (73/15)	32.3 ± 7.9 (21.8-55.1)	4.2 ± 1.7	62.9 ± 24.9	3.6 ± 1.2	1 bar (88), 2 bars (10)	Pneumothorax (11), pleural effusion (8), pneumonia (2), hemothorax (2), pulmonary embolism (1), wound infection (10), seroma (3), allergic reaction (2), reoperation (5), bar displacement (4), prolonged chest pain requiring narcotics for > 8 weeks (12), perioperative ventricular arrhythmia (1)
Viggiano et al. (2022) [16]	93 (85/8)	23* (18-42)	5.1 (2.3-12.6)	45* (35-95)	7* (5-13)	1 bar (69), 2 bars (24)	Seroma/hematoma (2), wound infection (2), hemothorax (1), pneumothorax (4), bar displacement requiring reoperation (4)
Wang et al. (2021) [17]	100 (79/21)	22.03	4.1	64.94	5.77	1 bar (87), 2 bars (13)	Pneumothorax (2), pleural effusion (1), atelectasis (3), wound infection (2), bar displacement (2), bar exposure due to delayed wound healing (2)

Of the 10 studies included in this review, five compared complications with patients in different age groups [9-10,12-14]. In all five of these studies, patient age correlated with complication rate, as older patients suffered more complications than their younger counterparts. de Loos et al. found that patients older than 24 years had significantly higher postoperative chronic pain and a higher use of two bars compared to patients younger than 24 [9]. Erşen et al. found that in adults over the age of 18, there was a significantly higher rate of complications, specifically displacement of the steel bar, compared to patients under 18 [10]. Jaroszewski et al. compared outcomes in two cohorts of patients with one 18-29 years old and the other ≥ 30 years old. In addition to the Nuss procedure, 31.6% of patients also required open cartilage resection, sternal osteotomy, or both, with this modification being more common among patients ≥ 30 years old. Patients requiring this modification within the ≥ 30 years old group were older (47.8 ± 13.1 years versus 39.5 ± 7.8 years) and had a higher mean preoperative Haller index (7.7 ± 5.6 versus 5.5 ± 3.2) than patients who did not require it. Additionally, patients ≥30 had significantly higher instances of pneumonia, urinary tract infections, ileus, hospital readmissions, and bar displacement than those between 18 and 29 [12]. Kim et al. found that patients older than 19 years had a significantly higher rate of bar displacement and wound infection due to the stabilizer, both requiring reoperation, compared to patients younger than 19 [13]. Lo et al. compared outcomes in three groups of patients ranging from 12-19 years, 19-30 years, and > 30 years. Preoperative symptoms, including exertional dyspnea, chest pain, palpitations, significant chest deformity, anxiety/depression, and valvular regurgitations, were significantly more common in patients > 30. Additionally, patients > 30 had a significantly longer length of operation compared to the two other groups. Peri- and postoperative complications were, however, more dependent on the preoperative Haller index rather than patient age [14].

Discussion

There is significant debate regarding the use of the Nuss procedure to correct PE in adult patients since it is commonly indicated for patients in the pediatric population. This review provides a compilation of complications and outcomes of the Nuss procedure in adult patients without any prior intervention for PE. The findings suggest that postoperative complications are more common in adults, with a higher incidence in older adults compared to young adults. The most common complications observed in adult patients were bar displacement, wound infection, pneumothorax, pleural effusion, reoperation, and chronic pain. Despite complications being more common with increased age, nine of 10 articles included in this review recommend the use of either a traditional or modified Nuss procedure to correct PE deformities in adult patients [8-11,13-17]. Kim et al. reported significantly high rates of dissatisfaction in adult patients, mainly due to a higher incidence of preoperative asymmetrical deformity, which increased the risk of complications. The most common complication with asymmetrical deformities included a higher incidence of bar displacement. Adults with symmetrical deformities, however, had risks of complications and outcomes comparable to their pediatric and adolescent counterparts, highlighting the importance of appropriate patient selection for the Nuss procedure [12].

The original procedure described by Nuss utilized one bar in the majority of cases, with two being used in situations requiring additional stabilization due to more severe deformities [6]. This technique has since been slightly modified in some cases with the use of shorter and more bars, allowing for greater stabilization without additional sutures, a reduced rate of bar displacement, and easier guidance through the chest using only the surgeon’s fingertip [18]. Despite these modifications, bar displacement remains a major postoperative complication which in many cases requires reoperation. The ideal candidates for the Nuss procedure have traditionally been children and adolescents because their sternum is still very elastic, allowing for a much easier sternal elevation without the need for multiple bars. Adult patients however have a much larger and more rigid sternum, which places them at higher risk for complications, particularly bar displacement and chronic pain, due to increased pressure placed on their chest with the placement of the sternal bar [19]. Sa et al. found that a combination of forced sternal elevation and video-assisted thoracoscopy during the placement of the bar resulted in lower incidences of bar displacement. Even with this technique, age and a higher preoperative Haller index directly correlated with the severity of bar displacement in addition to the overall rates of bar displacement [20]. Additionally, excessive sternal rigidity in adults is also most likely why they commonly require multiple bars compared to pediatric patients, which can explain their observed higher mean operating times and rate of postoperative infections. In our review, 138 (12.2%) patients had three or four bars implanted, with an increase in the number of bars being more common amongst older patients.

In the three patients that required conversion to a modified Ravitch procedure due to severe asymmetry, it was determined that a Nuss procedure would not suffice to correct the PE deformity [10,12]. In patients with a severe deformity, an open technique, such as the modified Ravitch procedure, is preferred due to the ability to fracture the sternum at various locations and resect the involved costal cartilage while preserving the perichondrium, all based on the unique asymmetry of each patient. This allows for a higher degree of correction, decreasing the risk of recurrent PE, yet at the cost of a longer operation time and a significantly larger scar on the anterior chest [21]. Because many asymptomatic patients undergo surgical repair for PE due solely to cosmetic reasons, it is vital to thoroughly assess each patient to determine appropriate candidates for the Nuss procedure compared to other interventions, which may better suit the patient’s needs [10]. If a patient decides to undergo correction of PE solely for cosmetic reasons, novel minimally invasive options, such as custom-made silicone implants, exist to fulfill the patient's needs. This approach is, however, risky and long-term results have not yet been assessed [22]. Proceeding with a Nuss procedure in a patient with severe asymmetry may increase the risk for recurrent PE, requiring the need for further reoperations, which may place the patient at an increased risk for peri- and postoperative complications.

There has been much debate regarding the negative impact of PE on cardiopulmonary function, however, recent studies have provided growing evidence of an existing relationship. Gürkan et al demonstrated significant improvement in right ventricular function parameters following the Nuss procedure. Preoperatively, right ventricular compression was observed due to PE via Doppler echocardiography, highlighting the impact of PE on cardiac function [23]. Many patients elect to undergo the Nuss procedure due to cardiopulmonary deficits which are exacerbated during exercise, however, the impact of the Nuss procedure on cardiopulmonary function has not been studied in as much detail in adults compared to pediatric patients. Jaroszewski et al. found that in adult patients undergoing the Nuss procedure, significant improvements in cardiopulmonary outcomes were seen, including increased maximum and predicted rate of oxygen consumption, oxygen pulse, oxygen consumption at anaerobic threshold, and maximal ventilation [3]. Neviere et al. similarly found that at the one-year follow-up of the Nuss procedure, adult patients had sustained significant improvement in cardiopulmonary function observed during exercise. Specifically, increased postoperative maximal oxygen uptake during exercise suggested that the Nuss procedure allowed for better cardiovascular adaptation at maximal workload [24]. Growing evidence of cardiopulmonary improvement following the Nuss procedure highlights both preoperative cardiopulmonary deficits due to PE as well as the effectiveness of PE repair on cardiopulmonary outcomes.

## Conclusions

In patients with no prior intervention for PE, the overall rate of complications is higher in adults compared to children and adolescents undergoing the Nuss procedure, with bar displacement being the most common complication. Additionally, within adult groups, older adults have a higher rate of complications compared to young adults. Despite evidence that the rate of complications increases with age, the majority of adult patients in our included studies were satisfied with the outcome of the procedure, with indications of improved self-image and reduced preoperative symptoms such as dyspnea on exertion, palpitations, chest pain, and depression. Unfortunately, few studies examining complications and long-term outcomes in adult patients exist, making it difficult to derive firm conclusions regarding the success of the Nuss procedure in adult patients.
